# Clinical Data and Health Outcomes for HIV-Positive Patients Diagnosed With COVID-19

**DOI:** 10.7759/cureus.22342

**Published:** 2022-02-17

**Authors:** Ayoola Adigun, Farouk Meklat, Diana Brown

**Affiliations:** 1 Grants Administration, Broward Health Medical Center, Fort Lauderdale, USA; 2 Infectious Disease, Broward Health Medical Center, Fort Lauderdale, USA; 3 Comprehensive Care Center, Ryan White Parts A & C, Broward Health Medical Center, Fort Lauderdale, USA

**Keywords:** cd4+, pandemic, epidemiology and biostatistics, hiv viral load, retrospective research

## Abstract

Introduction

As we care for patients during the coronavirus pandemic caused by severe acute respiratory syndrome coronavirus 2 (SARS-CoV-2), it is important to learn and analyze the health outcomes for HIV-positive patients who have been infected with COVID-19. The clinical course and outcome of COVID-19 among patients with HIV-1 infection are still unknown and novel.

Methods

This is a retrospective cohort study of 34 HIV-positive patients who are diagnosed with COVID-19. The following basic demographic, clinical, and laboratory test information were collected for each patient: age, race/ethnicity, gender, CD4/viral load count before and after COVID-19 diagnosis, clinical symptoms, hospitalizations, antiretroviral medications, and comorbidities. These data were collected from the electronic health record (EHR) and recorded in the study database.

Results

The mean (interquartile range (IQR)) HIV viral load (RNA PCR) after COVID-19 infection was 37,170 (<20-167) copies/mL compared to 25,730 (<20-100) copies/mL before COVID-19 infection. The mean (IQR) CD4+ lymphocyte count prior to and after COVID-19 infection was 583 (101-1139) and 477 (167-821) cells/mm^3^, respectively. Hypertension (n = 20) was the most prevalent comorbidity found in the cohort of HIV-positive patients. Patients with HIV RNA < 20 copies/mL prior to and after COVID-19 infection were 27 (79.3%) and 17 (73.7%), respectively.

Conclusion

As the pandemic situation keeps on evolving, there will be new findings on how people living with HIV might be affected by SARS-CoV-2. Our findings highlight the importance of larger sample size studies to better understand the management of HIV-positive patients in a pandemic situation.

## Introduction

There has been evolving and conflicting evidence whether people living with HIV have an increased risk of acquiring severe acute respiratory syndrome coronavirus 2 (SARS-CoV-2) infection compared to the general population [1]. One of the first known cases of COVID-19 infection in an HIV-positive patient was described in Wuhan City, China [2,3]. The HIV/AIDS epidemic was first described in 1981 [4], and since then, the medical community has made advancements in understanding its pathogenesis, prevention, and treatment to curb its progression. In the early days of the SARS-CoV-2 pandemic, we found that the effects of COVID-19 on HIV-positive patients were still unknown as HIV is a chronic medical condition that will need to be studied further extensively going forward.

The clinical course and epidemiology of SARS-CoV-2 and HIV coinfection are still being established. The World Health Organization (WHO) and Center for Disease Control and Prevention (CDC) have issued health alerts and prevention guidelines for people at increased risk for severe health outcomes and mortality due to COVID-19 [5,6]. Patients who have chronic disease comorbidities have been identified to have an increased risk for SARS-CoV-2 morbidity and mortality [7]. It has been studied that the immunodeficiency caused by chronic HIV infection increases the risk of coinfection with pathogens that are not controlled by innate and adaptive cellular responses and some that are controlled by phagocytic antibody responses [8]. Additionally, the administration of combination antiretroviral therapy (ART) during HIV coinfection does not always restore the pathogen-specific immune response to normal levels, which further increases a patient’s susceptibility to poor health outcomes, and in our case, patients might have a poor prognosis if they contract COVID-19 [8].

Moghadas et al. found that vaccination can substantially impact COVID-19 outbreak mitigation, even with limited protection against infection, but to achieve this, it is essential to continuously comply with nonpharmaceutical interventions [9]. According to the Joint United Nations Programme on HIV/AIDS (UNAIDS), 26 million people were accessing antiretroviral therapy (ART) as of the end of June 2020 and 38 million people globally are living with HIV in 2019 [10]. To the best of our knowledge, there exists limited information on the clinical outcomes of HIV-positive hospitalized patients who are infected with COVID-19. In this report, we aim to describe the clinical course and health outcomes as it relates to what type of ART they have been prescribed. We ascertain if there is a trend of those contracting the virus being on the same class of antiretroviral medication, and we also examine the patient’s HIV viral load (RNA PCR) and CD4/CD8 count to determine if there is any correlation with those being virally suppressed or not.

## Materials and methods

Study population and setting

This is a retrospective cohort study of patients who have been diagnosed with HIV and then got diagnosed with SARS-CoV-2. Retrospective data collection was done at Broward Health Medical Center, Fort Lauderdale, USA. A confirmed case of COVID-19 was defined by a positive real-time reverse-transcription PCR (RT-PCR) assay for SARS-CoV-2 on a nasopharyngeal swab and/or positive serology for SARS-CoV-2 (positivity of immunoglobulin (Ig) G, M, or A). The eligibility criteria include patients who are HIV-positive (diagnosis done using standard laboratory essays) and adults aged 18 years and older.

Patients at our hospital institution are evaluated for inpatient admission based on the following criteria: severe dyspnea, oxygen saturation on room air of ≤90% regardless of the severity of dyspnea, and concerning alterations in mentation (e.g., confusion, change in behavior, and difficulty in rousing) or other signs and symptoms of hypoperfusion or hypoxia (e.g., falls, hypotension, cyanosis, anuria, and chest pain suggestive of an acute coronary syndrome). Patients meeting the above criteria will typically be admitted to the hospital for inpatient evaluation and management. Patients were hospitalized according to the severity of their symptoms on presentation, such as dyspnea, fever, cough, myalgia, headache, new loss of taste or smell, sore throat, nausea/vomiting, nasal congestion, and diarrhea. The patients’ medical history was taken, and they were asked if they had ever come in contact with a known positive COVID-19 patient. Demographic baseline characteristics collected for each patient include birth date, race/ethnicity, and gender. We also collected data on CD4+ lymphocyte count, HIV viral load, HIV RNA < 20 copies/mL, comorbidities, antiretroviral medication, and past medical history.

Statistical analysis

Descriptive statistics were done for categorical and continuous variables. Categorical variables were presented as frequencies (n) and percentages (%). Continuous variables were presented as means and interquartile ranges (IQRs). A bar chart distribution was done to show the mean distribution of CD4+ lymphocyte count (cells/mm^3^) and HIV viral load (RNA PCR) before and after COVID-19 infection. All analyses were performed using STATA/IC version 16.1 for Windows (StataCorp LLC, College Station, Texas, USA).

Ethics

All authors had access to the study data, reviewed, and approved the final manuscript. There are no issues related to consent, and the study period was from May 1, 2020, to December 31, 2020; during this period, electronic medical records were reviewed retrospectively.

## Results

During the study period, we identified 34 patients who had a coinfection of HIV and SARS-CoV-2. Males were 61.8% (21) and females 38.2% (13). The median age of patients in the study was 54 years. African Americans constituted the majority of the population at 76.5%, Hispanics at 2.9%, and Caucasians at 20.6%. The HIV baseline status of the patients showed a mean higher HIV viral load of patients before and after being diagnosed with COVID-19. The mean HIV viral loads (RNA PCR) before and after COVID-19 infection were 25,730 and 37,170 copies/mL, respectively. The mean CD4+ lymphocyte count before COVID-19 infection was higher compared to after being diagnosed with COVID-19, with values of 583 and 477 cells/mm^3^, respectively (Table 1). There were 27 patients with HIV RNA < 20 copies/mL prior to COVID-19 infection and 17 patients with HIV RNA < 20 copies/mL after COVID-19 infection (Table 1).

The most common comorbidities include hypertension (58.8%) and type II diabetes mellitus (32.3%). The least common comorbidities were end-stage renal disease (2.9%), congestive heart failure (5.9%), and obesity (8.8%) (Table 1). Two patients died during hospitalization and one outside the hospital after discharge. The first patient went into hypoxic respiratory failure and cardiopulmonary arrest, which required intubation. The second patient passed away from an unknown cause, while the third patient died from complications related to his diabetes outside the hospital.

We had two patients who can be considered outliers as regards their HIV viral load (RNA PCR). The first patient had a viral load of 742,273 copies/mL before COVID-19 infection and was just recently diagnosed. The second patient had a viral load of 482,000 copies/mL after COVID-19 infection.

**Table 1:** Demographic data and clinical characteristics of patients with COVID-19 and HIV infection

Variables	
Patients, n	34
Demographics	
Male, n (%)	21 (61.8)
Female, n (%)	13 (38.2)
Age (years), median (IQR)	54 (47–61)
Race/ethnicity	
Black, n (%)	26 (76.5)
Hispanic, n (%)	1 (2.9)
White, n (%)	7 (20.6)
HIV baseline status	
HIV viral load (RNA PCR) prior to COVID-19 infection, mean (IQR)	25,730 (<20–100)
HIV viral load (RNA PCR) after COVID-19 infection, mean (IQR)	37,170 (<20–167)
CD4+ lymphocyte count (cells/mm^3^) prior to COVID-19 infection, mean (IQR)	583 (101–1,139)
CD4+ lymphocyte count (cells/mm^3^) after COVID-19 infection, mean (IQR)	477 (167–821)
Patients with HIV RNA < 20 copies/mL prior to COVID-19 infection, n (%)	27 (79.3)
Patients with HIV RNA < 20 copies/mL after COVID-19 infection, n (%)	17 (73.7)
Comorbidities, n (%)	
Asthma	3 (4.8)
Congestive heart failure	2 (3.2)
Coronary artery disease	4 (6.5)
Deceased	3 (4.8)
Diabetes mellitus	11 (17.8)
Prediabetes	3 (4.8)
End-stage renal disease	1 (1.6)
Hyperlipidemia	8 (12.9)
Hypertension	20 (32.3)
Obesity	3 (4.8)
Pneumonia	4 (6.5)

Biktarvy tablets (bictegravir, emtricitabine, and tenofovir alafenamide) were the most common drug used in our cohort of patients (33.4%), followed by Symtuza (darunavir, cobicistat, emtricitabine, and tenofovir alafenamide) (23.3%). Genvoya (elvitegravir, cobicistat, emtricitabine, and tenofovir alafenamide) (3.3%), Odefsey (emtricitabine, rilpivirine, and tenofovir alafenamide) (3.3%), and Triumeq (abacavir, dolutegravir, and lamivudine) (3.3%) were the least used ARTs found among our patients.

**Figure 1:**
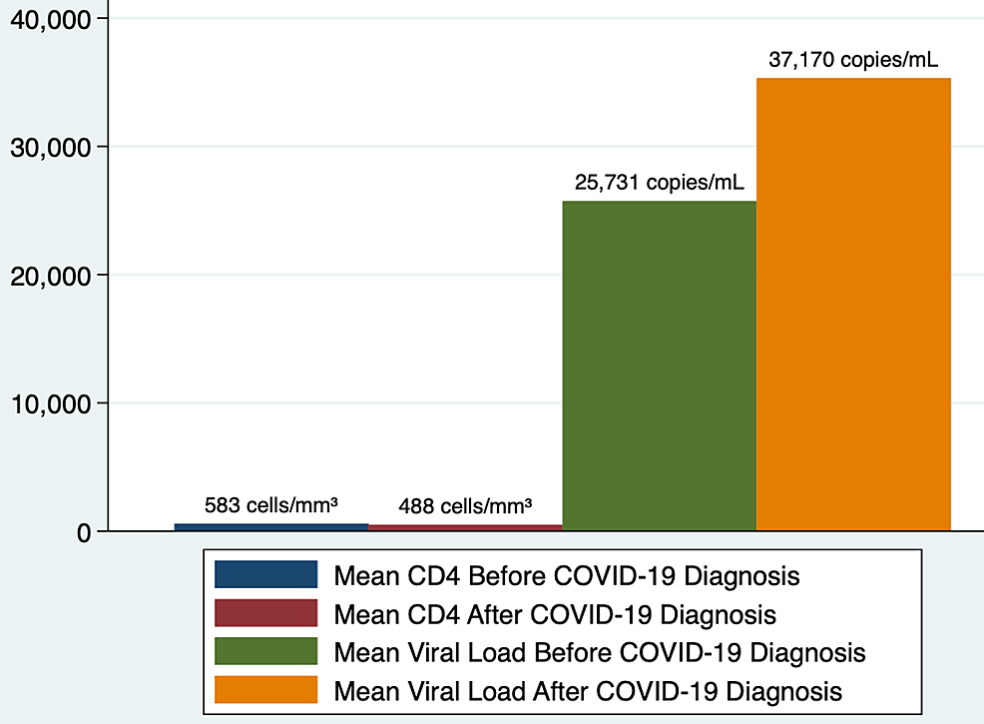
Mean distribution of CD4+ lymphocyte count (cells/mm³) and HIV viral load (RNA PCR) before and after COVID-19 infection

Figure 1 depicts the bar chart showing the mean CD4+ lymphocyte count (cells/mm^3^) and HIV viral load (RNA PCR) distribution before and after diagnosis of COVID-19 infection in our cohort of patients. CD4+ ranges from 18 to 1,335 cells/mm^3^, and we defined immunosuppression at CD4+ < 200 cells/mm^3^, which can lead to opportunistic infections [11].

## Discussion

This is a descriptive study with no cause-effect relationship or association between variables. In our study of 34 HIV-positive patients who had coinfection with SARS-CoV-2, we found a trend in the distribution of their mean CD4+ lymphocyte count and HIV viral load. The HIV-positive patients who became infected with COVID-19 had a lower mean CD4+ lymphocyte count compared to prior COVID-19 infection. Also, the HIV-positive patients who became infected with COVID-19 had a higher mean HIV viral load (RNA PCR) compared to prior COVID-19 infection. The etiology and pathogenesis for this particular trend found in our cohort of patients need to be further investigated. During our study, COVID-19 seems not to affect or interrupt the course of our patient’s HIV treatment, and their treatment was followed accordingly.

A study found that people who live with HIV that is well-controlled were not at a higher risk for poorer COVID-19 outcomes than the general population [12]. However, our findings cannot be generalized or extrapolated to describe the patient health outcomes of HIV-positive patients who contract COVID-19. This is due to the limited sample size, unique characteristics of the patients, and geographic location. When the COVID-19 pandemic started, there was limited information and knowledge to understand how it might affect people with a predisposition to immunosuppression such as those with HIV who are not medically compliant. We are still learning about COVID-19, and although the clinical characteristics of COVID-19 have been broadly described [13], there is still scarce literature on the clinical features including comorbidities, presenting symptoms and signs, severity, complications, and outcomes among HIV-positive persons who become infected with COVID-19. There are also uncertainties regarding the laboratory parameters that are predictive of disease severity and outcome of COVID-19 among people living with HIV. With 37.9 million people living with HIV and 1.7 million new infections each year [14], patients of COVID-19 and HIV coinfection are likely to increase. The amount of information available that has been firmly established regarding the extent to which persons living with diagnosed HIV are acquiring COVID-19, the severity of COVID-19 illness experienced by persons living with diagnosed HIV, or how these distributions compare with persons without diagnosed HIV remains minimal.

Limitations

This study has limitations such as lack of generalizability as the results do not represent the nationwide trends. This can be attributed to the fact that the study was conducted at a single medical center. Furthermore, our study is limited by its retrospective study design, which depends on data that was already entered into a clinical database and not collected for research. Since the data was not collected in a predesigned proforma as per the specific requirements of the study, some data will inevitably be missing in most of the cases. Also, certain variables that have the potential to impact the outcome may not have been recorded at all. Another limitation worth mentioning in our study is the use of secondary data collection, which makes our study team not privy to information about how seriously the data is affected by problems such as low response rate or respondent misunderstanding of specific survey questions.

## Conclusions

Our study sought to explore the clinical course and outcome(s) of COVID-19 among patients with HIV-1 infection, which are still novel and largely unknown. We found that most patients who became infected with COVID-19 and were subsequently hospitalized were diagnosed with either hypertension or type II diabetes mellitus. There need to be large-scale prospective cohort studies that will generate definite results about the causal relationship/association between a patient’s HIV baseline status and how it might predispose them toward HIV/COVID-19 coinfection. This will help us prevent and reduce the future threat of pandemics in HIV-positive patients and further understand how it affects morbidity and mortality rates in these patients.
